# 多发性骨髓瘤双特异性抗体治疗相关感染管理中国专家共识（2026年版）

**DOI:** 10.3760/cma.j.cn121090-20260211-00088

**Published:** 2026-06

**Authors:** 中华医学会血液学分会浆细胞疾病学组 中国医师协会多发性骨髓瘤专业委员会

## Abstract

双特异性抗体（BsAb）是治疗复发/难治性多发性骨髓瘤（MM）的新型免疫疗法，通过连接T细胞与肿瘤细胞表面抗原诱导肿瘤杀伤，显著延长了患者的生存期。然而，BsAb独特的免疫激活机制导致持续T细胞激活、B细胞耗竭及低丙种球蛋白血症，使患者感染风险明显增加，总体感染发生率为45.8％～80％，已成为影响治疗安全性的关键问题。随着BsAb在我国的广泛应用，对其相关感染的认识不断加深，感染监测技术、预防方案及治疗策略亦在持续优化。为规范我国MM患者接受BsAb治疗相关感染的防控实践，中华医学会血液学分会浆细胞疾病学组与中国医师协会多发性骨髓瘤专业委员会基于最新证据制定了本版共识。本共识主要适用于MM患者接受BsAb治疗过程中的感染监测、预防与处理，提升对感染相关并发症的识别与管理能力，使患者获得最佳的无进展生存期和总生存期。

## Abstract (en)

Bispecific antibody（BsAb）is a novel immunotherapy for relapsed/refractory multiple myeloma（MM）, which induces tumor cell killing by bridging T cells with tumor surface antigens and has significantly prolonged patient survival. However, the unique immune activation mechanism of BsAb leads to sustained T-cell activation, B-cell depletion, and hypogammaglobulinemia, markedly increasing infection risk in patients. The overall incidence of infection is 45.8％–80％, becoming a critical issue affecting treatment safety. With the widespread application of BsAb in China, understanding of related infections has been continuously deepened, and infection monitoring techniques, prevention protocols, and treatment strategies are also being optimized. To standardize the prevention and control of BsAb-related infections in MM patients in China, the Plasma Cell Disease Group, Chinese Society of Hematology, Chinese Medical Association and Chinese Myeloma Committee-Chinese Hematology Association have developed this consensus based on the latest evidence. This consensus mainly applies to the infection monitoring, prevention, and management during the treatment of BsAb in MM patients, aiming to enhance the recognition and management capability of infection-related complications and enable patients to achieve optimal progression-free survival and overall survival.

多发性骨髓瘤（multiple myeloma，MM）是一种恶性浆细胞疾病，为血液系统中第二常见的恶性肿瘤。近20年，MM新药不断研发，患者生存期显著延长，但由于疾病本身的特点（患者免疫功能受损、高龄、治疗相关因素等），感染仍是MM早期死亡的主要原因之一，是影响生存不容忽视的问题[1]–[2]。因此，防治感染是贯穿MM治疗全过程的重要话题。双特异性抗体（bispecific antibody，BsAb）作为一种新型免疫疗法，通过连接免疫效应细胞表面的受体与肿瘤细胞表面的特异性抗原高效诱导肿瘤杀伤，表现出深度且持久的反应，显著延长了复发/难治性MM（relapse and refractory multiple myeloma，RRMM）患者的整体生存期[3]。现有2个靶点的BsAb展现出显著的疗效，但其独特的免疫激活机制带来的持续T细胞激活、T细胞和（或）B细胞耗竭等也会引起严重免疫抑制，导致患者感染风险明显增加[4]–[6]。BsAb总体感染发生率为45.8％～80％，其中靶向B细胞成熟抗原（B-cell maturation antigen，BCMA）的发生率可高达80％，3～4级感染发生率为20.3％，治疗中断率高，严重影响BsAb在MM中的广泛应用[6]–[7]。因此，BsAb相关的感染防控已成为MM患者整体感染防控体系中需关注的核心问题之一。充分认识BsAb治疗相关感染风险，做好监测、诊断及合理使用抗感染药物对于降低BsAb治疗后感染的发生率和病死率至关重要。本文重点围绕MM患者接受BsAb治疗过程中的感染风险、监测、预防及处理提出建议，旨在为MM临床实践提供更具针对性的参考。需要说明的是，本共识主要依据目前MM领域BsAb治疗相关研究证据及临床实践形成，适用对象为MM患者；对于其他血液系统恶性肿瘤中BsAb治疗相关感染管理问题，尚有待更多针对性证据支持。

一、共识制订方法

1. 共识目的：本共识旨在结合国内外最新研究证据、药品安全性资料及我国临床实践经验，规范MM患者接受BsAb治疗过程中感染的风险识别、监测、预防及处理策略，提高临床医师对BsAb治疗相关感染并发症的识别和管理能力，为我国MM患者接受BsAb治疗期间的感染防控提供参考。

2. 共识制订专家组成：本共识的制订工作组由国内具有MM诊疗和感染管理经验的血液科专家组成。制订工作组成员按照主要职能分为通信作者、执笔专家和共识讨论专家。所有制订工作组成员均填写了利益冲突声明表，均声明不存在与本共识相关的经济或非经济利益冲突。制订过程或推荐意见形成未受到资助影响。

3. 共识注册：本共识在制订初期撰写了计划书，并在国际实践指南注册与透明化平台（http://www.guidelines-registry.cn）进行了中文注册（注册号：PREPARE-2025CN1603）。

4. 共识目标人群与使用者：本共识的目标人群为接受或拟接受BsAb治疗的MM患者，尤其是RRMM患者。

本共识的主要使用者为从事MM诊疗及感染管理的血液科医师，也可供感染科、呼吸科、重症医学科医师，临床药师，护理人员及参与患者随访管理的相关医务人员参考。

5. 临床问题及共识内容的确定：本共识未按照正式循证临床实践指南的方法学流程预先构建PICO问题，也未采用问卷调查或德尔菲法对临床问题进行独立遴选。考虑到BsAb治疗MM相关感染管理目前仍缺乏高质量前瞻性研究证据，且临床实践中亟需解决的问题主要集中于治疗前评估、治疗期间监测、感染预防和感染处理等连续管理环节，专家组根据现有文献证据、药品说明书、安全性数据及我国临床实践经验，按照BsAb治疗相关感染管理的实际流程确定共识内容框架。

具体而言，专家组围绕以下临床实践中常见且具有重要管理价值的问题展开讨论和撰写：①BsAb治疗MM相关感染的流行病学特征及主要危险因素；②治疗前需要完成的感染筛查项目；③治疗期间无症状感染患者及疑似感染患者的监测和诊断策略；④细菌、病毒、真菌、结核分枝杆菌等感染的预防措施；⑤低丙种球蛋白血症（hypogammaglobulinemia，HGG）患者免疫球蛋白替代治疗的适用情形；⑥感染发生后的抗感染治疗原则及BsAb暂停、恢复或终止治疗的处理策略。

因此，本共识中的临床问题并非以独立PICO问题形式呈现，而是嵌入感染管理全流程的章节结构中。专家组认为，该结构更符合BsAb治疗MM相关感染管理的临床决策路径，也便于临床医师在实际工作中查阅和应用。

6. 文献检索与资料来源：为支持共识内容撰写，专家组对BsAb治疗MM相关感染的研究证据和感染管理资料进行了文献检索和整理。检索数据库主要包括PubMed、Embase、The Cochrane Library、Web of Science、中国知网、万方数据库和维普数据库等，检索时间范围为建库至2025年12月。

英文检索词主要包括：“multiple myeloma”、“relapsed/refractory multiple myeloma”、“bispecific antibody”、“bispecific antibodies”、“BCMA”、“GPRC5D”、“FcRH5”、“teclistamab”、“elranatamab”、“talquetamab”、“infection”、“infectious complications”、“hypogammaglobulinemia”、“intravenous immunoglobulin”、“Pneumocystis jirovecii pneumonia”、“cytomegalovirus”、“hepatitis B virus”、“tuberculosis”、“vaccination”、“antimicrobial prophylaxis”等。

中文检索词主要包括：“多发性骨髓瘤”、“复发/难治性多发性骨髓瘤”、“双特异性抗体”、“双抗”、“感染”、“感染并发症”、“低丙种球蛋白血症”、“静脉注射免疫球蛋白”、“耶氏肺孢子菌肺炎”、“巨细胞病毒”、“乙型肝炎病毒”、“结核”、“疫苗接种”、“抗感染预防”等。

资料来源包括国内外BsAb治疗MM的关键临床研究、真实世界研究、安全性分析、药品说明书及血液肿瘤、免疫治疗、细胞治疗和感染防控相关指南、共识及专家建议。对于证据相对充分且国内外意见较一致的内容，专家组优先参考临床研究、药品说明书及既往指南/共识；对于证据有限或尚无统一标准的问题，则结合我国临床实践经验进行讨论后形成共识性意见。

7. 共识建议的形成：本共识中的建议并非基于正式证据分级和推荐强度评价形成，而是由专家组在充分讨论现有研究证据、药品说明书、既往指南/共识及我国临床实践经验的基础上形成。对于证据较为明确且临床可操作性较强的内容，如治疗前病毒筛查、耶氏肺孢子菌肺炎（pneumocystis jirovecii pneumonia，PJP）预防、乙型肝炎病毒（HBV）再激活管理、免疫球蛋白水平监测及静脉注射免疫球蛋白（intravenous immunoglobulin G，IVIG）替代治疗等，专家组结合我国临床可及性和患者特点提出相应共识建议。

对于目前证据尚不充分或仍存在争议的内容，如是否进行常规细菌预防、是否进行常规侵袭性真菌感染预防、巨细胞病毒（cytomegalovirus，CMV）预防性治疗及疫苗接种时机等，本共识采用较为审慎的表述，强调应结合患者个体感染风险、免疫抑制程度、既往感染史、当地病原学流行情况及医疗机构诊疗规范进行综合判断。

共识文本由执笔专家根据章节分工完成初稿撰写，经专家组多轮审阅、讨论、修改和补充后形成终稿。对于存在争议的问题，经专家组充分讨论后，以多数专家认可、临床可操作性较强且安全性优先为原则进行表述。

8. 局限性与更新：目前BsAb在MM中的应用仍处于快速发展阶段，相关感染管理策略主要来源于关键临床研究、安全性分析、真实世界研究、药品说明书及专家经验，尚缺乏针对我国人群的大规模前瞻性研究证据。由于本共识未开展系统评价和推荐强度分级，文中建议不代表按照GRADE或其他证据分级体系形成的正式推荐意见，临床应用时应结合患者个体情况、医疗机构条件及当地感染病原谱进行判断。

随着BsAb药物种类、适应证、联合治疗方案及真实世界感染数据不断积累，本共识将根据新的临床证据和我国实践经验适时更新。

二、流行病学

1. BsAb治疗MM相关感染的流行病学特征：现有临床数据显示，BsAb治疗MM相关感染总体发生率较高（表1）[4],[8]。Cani等[9]纳入158例接受BsAb治疗的RRMM患者，其中靶向BCMA的BsAb组第5个月和10个月时首次任何级别感染累积发生率分别为38.6％和47.9％，靶向G蛋白偶联受体C类第5组成员D（GPRC5D）的BsAb组分别为28.1％和30.3％。研究显示，感染可影响患者治疗的疗程，导致患者治疗延迟或中断甚至死亡[6]。在11项BsAb相关的临床试验中，110例患者死亡，其中26％与感染并发症相关[10]。

**表1:** 中国已获批的双特异性抗体关键研究中感染发生率[8]

药物	研究	靶点	例数	中位随访时间（月）	1～4级感染发生率	≥3级感染发生率	1～4级感染例数^a^	≥3级感染例数^a^
特立妥单抗	MajesTEC-1	BCMA-CD3	165	22.8	80.0％	55.2％	3.5	2.4
埃纳妥单抗	MagnetisMM-1	BCMA-CD3	55	12.0	74.5％	27.3％	6.2	2.3
埃纳妥单抗	MagnetisMM-3	BCMA-CD3	123	14.7	69.9％	39.4％	4.8	2.7
塔奎妥单抗（0.4 mg/kg）	MonumenTAL-1	GPRC5D-CD3	143	14.9	58.0％	22.0％	3.9	1.5
塔奎妥单抗（0.8 mg/kg）	MonumenTAL-1	GPRC5D-CD3	145	14.9	65.0％	16.0％	4.4	1.1

**注** BCMA：B细胞成熟抗原；GPRC5D：G蛋白偶联受体C类第5组成员D；^a^每月每100例患者中感染例数

不同的病原体感染发生的中位时间有差异。总体来说，自开始接受BsAb治疗至感染发生的中位时间为44～49 d[6],[11]，3～4级感染发生的中位时间为3.8个月[12]。常见病原体包括细菌、病毒和真菌[12]–[13]，主要累及呼吸道，其次是胃肠道、尿道和皮肤[6]。BsAb治疗后感染的病原微生物具有以下特点：

（1）细菌感染：中位发生时间为20～55 d[14]–[15]，多为革兰氏阴性菌，特别是肠杆菌（52％），其他致病菌包括铜绿假单胞菌、厌氧菌、肠球菌、葡萄球菌、链球菌属等[6]。

（2）病毒感染：中位发生时间为26～31 d[14]–[15]，呼吸道病毒常见[6],[16]，其他病毒如CMV、腺病毒、疱疹病毒、新型冠状病毒也有报道[4],[17]。RRMM患者病毒感染再激活的风险很高。来自韩国的报道显示，49.2％的患者在接受BsAb治疗的58 d内出现CMV再激活，其中24.6％具有临床意义，需要干预[18]。

（3）真菌感染：中位发生时间为41 d，且严重程度均较高[14]。BsAb治疗后发生的真菌感染中，曲霉菌感染占75％，赛多孢子菌占12.5％，耶氏肺孢子菌占12.5％[6]。

（4）地方性病原体感染：我国结核病发病率排全球第4位（2024年）[19]，慢性乙型肝炎感染者占全球三分之一（2023年）[20]。接受BsAb治疗的患者还需监测和管理结核病和乙型肝炎，特别是免疫功能低下患者[4]。中山大学附属第一医院的临床实践表明，HBV的激活也较常见。目前已有特立妥单抗治疗后MM患者发生HBV再激活的报道[21]。

2. 感染的危险因素：所有接受BsAb治疗的MM患者需要高度警惕感染风险和发生情况，发生感染的危险因素包括患者相关因素、疾病相关因素、治疗相关因素和感染史等（表2）。

**表2:** 接受双特异性抗体治疗的多发性骨髓瘤患者感染危险因素[16],[22]–[24]

类型	内容
患者相关因素	年龄（≥75岁）；体能状态（ECOG评分>2分）；合并症（如慢性心力衰竭）；中性粒细胞绝对计数减少（<0.5×10^9^/L）和淋巴细胞计数减少（<1.0×10^9^/L）；低免疫球蛋白血症（IgG/IgA/IgM下降）
治疗相关因素	大剂量糖皮质激素使用（如甲泼尼龙>1 g/d或地塞米松40 mg，每周4次）；既往接受过造血干细胞移植；既往接受过三线及以上治疗、抗CD38单克隆抗体、双特异性抗体或CAR-T细胞治疗等
疾病相关因素	肿瘤负荷（ISS分期Ⅱ～Ⅲ期）；肾功能不全
感染史	既往因感染导致的住院史；既往DNA病毒感染史，包括VZV、CMV及HBV

**注** ECOG：美国东部肿瘤协作组；CAR-T细胞：嵌合抗原受体T细胞；VZV：水痘-带状疱疹病毒；CMV：巨细胞病毒；HBV：乙型肝炎病毒

**推荐意见**

1. BsAb治疗MM患者感染发生率高，且常发生于治疗早期，严重感染可影响治疗连续性并增加死亡风险，应全程重视感染风险管理。

2. BsAb治疗相关感染以细菌、病毒和真菌感染为主，呼吸道为最常见感染部位，临床应结合常见病原体谱及感染发生时序加强识别。

3. 在我国，接受BsAb治疗的MM患者应特别重视结核病和乙型肝炎的监测与管理，尤其是免疫功能低下患者；对HBV再激活风险应保持高度警惕。

4. 高龄、体能状态差、合并中性粒细胞减少或淋巴细胞减少、低免疫球蛋白血症、肾功能不全、肿瘤负荷高、既往多线治疗或有感染住院史患者应作为感染高风险人群重点管理。

三、感染监测

（一）治疗前筛查

在接受BsAb治疗前，推荐进行CMV聚合酶链反应（PCR）、人类免疫缺陷病毒（HIV）、HBV和丙型肝炎病毒（hepatitis C virus，HCV）的血清学检测。HIV、HBV、HCV血清学检测阳性患者应进行PCR检测。此外，可根据当地疫情发展和流行病学情况，考虑进行新型冠状病毒检测（PCR或抗原）。对于阳性患者，应根据机构指南进行治疗或采取二级预防措施[4]。建议在开始用药前1周内行肺部CT检查，如有阳性发现，可结合纤维支气管镜明确病原学。

（二）治疗期间的监测与诊断

1. 无症状感染患者常规监测：基线PCR阳性患者的CMV PCR，应根据病毒载量并结合临床判断结果，阳性患者建议每1～2周复查1次PCR。建议根据病毒载量的变化和患者的临床情况，由医师决定是否应用BsAb治疗。对于HIV、HBV、HCV血清学检测阳性患者，早期建议每个月复查1次PCR，稳定后至少每3个月复查1次PCR[4]。

2. 疑似感染患者全面感染检查：

（1）病史询问和体格检查：详细了解既往发生感染和抗菌药物使用情况、病原微生物耐药和定植情况，发现感染的高危因素和隐匿部位[25]。

（2）实验室检查：定期复查全血细胞计数、肝肾功能和电解质，降钙素原、C反应蛋白等感染相关指标对于诊断感染有提示意义，需动态监测[25]。

（3）病原学检查：包括培养（如血液、尿液、痰液和粪便培养等）、微生物涂片、血清学、宏基因组二代测序（metagenomic next-generation sequencing，mNGS）、病毒的PCR检测和结核分枝杆菌免疫学检查（如结核菌素试验、干扰素释放试验等）等[25]。

当疑似真菌感染时，应及时开展G试验和（或）GM试验检测。但应注意IgG型骨髓瘤及部分应用静脉丙种球蛋白患者可出现G试验假阳性[26]。

（4）影像学检查：影像学检查（如CT、PET-CT、B超和头颅MRI）可更深入地了解并确认感染程度，以评估肺炎、疑似结肠炎、憩室炎或腹部脓肿、肺真菌或脑曲霉菌感染等，或根据感染部位进行手术活检[22],[25],[27]。

**推荐意见**

1. BsAb治疗前建议常规进行CMV PCR及HIV、HBV、HCV血清学筛查；对相关检测阳性者，应进一步行PCR检测。可根据当地流行病学情况考虑新型冠状病毒检测。治疗前建议于1周内完成肺部CT检查；如有异常，必要时结合纤维支气管镜明确病原学。

2. 基线CMV PCR阳性患者治疗期间建议每1～2周复查；HIV、HBV、HCV血清学阳性患者治疗早期建议每月复查PCR，稳定后至少每3个月复查1次。

3. 病原学和影像学检查应根据临床表现合理选择，包括培养、病毒PCR、mNGS、结核相关检查以及CT、PET-CT、B超、MRI等；疑似真菌感染时应及时完善G试验和（或）GM试验。

四、感染预防

1. 治疗相关感染的预防：

（1）细菌感染的预防：常规不推荐进行细菌感染的预防[22]。

若患者具有细菌感染的其他危险因素，如存在细菌感染史/复发性细菌感染史、已知的病原体定植或长期中性粒细胞减少（≥7 d），可选择左氧氟沙星（替代药物：头孢地尼、阿莫西林克拉维酸钾）进行预防[4],[22],[27]。

（2）病毒感染的预防：对于接受BsAb治疗的患者，推荐在治疗前进行水痘-带状疱疹病毒和单纯疱疹病毒的预防，用药时间应贯穿整个治疗过程及停药后3个月，常用药物包括阿昔洛韦或伐昔洛韦[4]。

对于乙肝表面抗原（hepatitis B surface antigen，HBsAg）阳性或HBV-DNA阳性的患者，建议在治疗前至少1周开始服用抗病毒药物（如恩替卡韦、替诺福韦等），疗程建议至少持续至免疫治疗停药后12～18个月。目前，关于乙肝核心抗体（hepatitis B core antibody，HBcAb）阳性的患者是否需要预防性抗病毒治疗的数据有限，仍有一定争议，但本共识建议，这些患者可以进行预防治疗。

若患者启动BsAb治疗前HBV-DNA水平超过10^3^ IU/ml，需先进行抗病毒治疗，待HBV-DNA降至10³ IU/ml以下，方可启动BsAb治疗。因停药后有HBV再激活风险，建议停药后每3个月复查HBV-DNA和肝功能，若出现HBV再激活需积极至感染科就诊[28]–[29]。

仅在疑似CMV相关疾病（例如结肠炎、肺炎、肝炎）、无法解释的发热和（或）血细胞减少或其他感染高风险患者中通过PCR监测病毒载量，不推荐在无明确CMV相关器官疾病的情况下进行CMV预防性治疗[26],[28]。

（3）真菌感染的预防：常规不推荐进行真菌感染的预防[30]。

但由于接受BsAb治疗患者的PJP发生率较高，且PJP死亡率较高，建议所有接受BsAb治疗的患者进行PJP预防治疗[22],[26]。药物推荐复方磺胺甲噁唑（严重的肝肾功能不全、葡萄糖-6-磷酸脱氢酶缺乏症患者除外），建议于BsAb治疗开始时启动预防，持续至少6个月或至CD4^+^T淋巴细胞≥200个/mm^3^[22],[27]。

除PJP外，其他真菌预防的原则参照《血液病/恶性肿瘤患者侵袭性真菌病的诊断标准与治疗原则（第七次修订版）》[31]。

（4）结核分枝杆菌感染的预防：对于免疫功能缺陷的高危人群，建议开展结核分枝杆菌潜伏期感染预防性治疗[32]。

我国目前推荐的结核预防性治疗方案主要有6～9个月单用异烟肼每日方案，3个月异烟肼、利福喷丁联合间歇方案，3个月异烟肼、利福平联合方案，4个月单用利福平方案[32]。对于多药耐药或利福平耐药结核病患者的接触者，应接受每日服用左氧氟沙星连续6个月方案作为结核病预防性治疗[33]。若评估结核分枝杆菌处于活动期，不建议应用BsAb。我国抗结核治疗应在胸科医院/感染科专家的指导下进行。

（5）疫苗接种：可以根据具体情况酌情进行疫苗接种，如新型冠状病毒疫苗、季节性流感疫苗（密切接触者）、肺炎球菌和流感嗜血杆菌疫苗、水痘-带状疱疹病毒疫苗[4],[27],[34]–[35]。但需强调，BsAb治疗早期不建议接种灭活疫苗，以避免免疫反应不足。禁止使用减毒活疫苗，并保持社交距离，戴口罩，勤洗手[4],[28]。

2. 特殊情况下的感染预防：MM患者通常会出现继发性免疫缺陷，如HGG，BsAb治疗可延长并加重HGG，导致感染风险进一步增加[22]。IVIG预防治疗有助于降低BsAb治疗相关的严重感染（≥3级）风险，改善无感染生存期和总生存期[9],[36]。建议开始BsAb治疗前检查基线免疫球蛋白水平，治疗后每月定期复查，所有IgG≤4 g/L和（或）合并严重或反复感染患者接受IVIG替代治疗，每月重复1次，直到IgG>4 g/L[22]。由于IgG半衰期较长，且IgG型MM在MM患者中患病率较高，可能导致部分患者外周血中总IgG浓度高于4 g/L的治疗临界值。但该数值中可能包含大量由骨髓瘤细胞分泌的克隆性IgG，而非具有正常免疫功能的多克隆IgG，因此需同时检测并评估患者的IgA、IgM浓度，同时从总IgG浓度中减去克隆性IgG的数值[4]。当无法精确计算或获得多克隆IgG水平时，若患者出现严重或反复感染，且IgA和（或）IgM水平显著降低（如低于正常值下限），也可作为启动IVIG替代治疗的有力指征。

**推荐意见**

1. BsAb治疗期间常规不推荐进行细菌或其他真菌感染预防；但对有细菌感染史/复发性细菌感染史、已知病原体定植或长期中性粒细胞减少者，可考虑抗细菌预防治疗。

2. 所有接受BsAb治疗的患者均建议进行单纯疱疹病毒和水痘-带状疱疹病毒预防；HBsAg阳性或HBV-DNA阳性患者应在治疗前至少1周启动抗病毒治疗，HBcAb阳性患者可考虑预防性抗病毒治疗；治疗前HBV-DNA>10^3^ IU/ml者，应先进行抗病毒治疗后再启动BsAb治疗。

3. 不推荐在无明确CMV器官疾病的情况下常规进行CMV预防性治疗；建议所有接受BsAb治疗的患者进行PJP预防，至少持续6个月或至CD4^+^ T细胞恢复至≥200个/mm^3^。

4. 存在结核感染风险的患者可考虑结核预防性治疗，活动性结核患者不建议启动BsAb治疗。

5. 可根据具体情况酌情接种相关疫苗，但治疗早期不建议接种灭活疫苗，且禁止使用减毒活疫苗；对于IgG≤4 g/L且（或）合并严重或反复感染者，建议进行IVIG替代治疗。

五、感染治疗

对患者进行综合评估后，在充分考虑患者的基础状况（既往感染史、高危因素、感染部位、脏器功能和耐药危险因素）及当地病原微生物流行病学、药物抗菌谱和不良反应的基础上，应尽快应用抗菌药物进行经验性治疗，必要时进行多学科诊疗[37]。BsAb与强效CYP3A4诱导剂、强效CYP3A4抑制剂之间无明显的相互作用[28]，但考虑到患者疾病情况复杂，仍需注意药物的相互作用及患者的肾功能情况，并及时调整药物剂量。

感染停用BsAb的标准：3级感染需停药，4级感染考虑永久停药；3～4级发热伴中性粒细胞减少也需暂停药物。恢复用药的标准：感染改善至≤2级（特立妥单抗）或≤1级/基线水平（埃纳妥单抗）后可恢复，并遵循延迟给药恢复方案。继续治疗依据：感染性不良反应为1～2级时，在积极处理和密切监测下可不中断治疗。

1. 细菌感染的治疗：对于明确病原菌的患者，可根据所识别细菌和药敏结果采用窄谱抗生素治疗。革兰阴性菌的治疗推荐三代头孢类、β-内酰胺类/酶抑制剂复合制剂、碳青霉烯类[25]。针对产超广谱β-内酰胺酶肠杆菌科细菌（ESBL-producing Enterobacterales，ESBL-E），首选β-内酰胺类/酶抑制剂复合制剂（轻中度）或碳青霉烯类（重度）[25]。碳青霉烯类耐药的肠杆菌科细菌（carbapenem resistant Enterobacteriaceae，CRE）推荐头孢他啶-阿维巴坦、替加环素、多黏菌素类药物、氨曲南-阿维巴坦或亚胺培南-雷利巴坦等[38]。铜绿假单胞菌感染推荐β-内酰胺酶抑制剂复合制剂（如头孢他啶/阿维巴坦、头孢哌酮/舒巴坦等）、头孢菌素类（如头孢他啶等）、碳青霉烯类（如美罗培南等）[25]。革兰阳性菌的治疗推荐恶唑烷酮类、糖肽类和环脂肽类等[25]。对于无法明确病原体的患者，尤其是合并中性粒细胞减少症的患者，推荐使用广谱抗生素，如第三代头孢菌素或碳青霉烯类药物等[26]。患者选择抗生素视患者的危险因素、诊断和药物可及性情况综合判断，具体用药调整方案可参考《中国中性粒细胞缺乏伴发热患者抗菌药物临床应用指南（2020年版）》[25]及《BCMA双特异性T细胞接合器治疗多发性骨髓瘤中国临床专家共识（2025年版）》[26]。

抗菌药物治疗的疗程取决于感染的严重程度、基础疾病、抗菌药物的杀菌作用等多方面因素[28]。一般宜用至体温正常、症状消退后72～96 h，但血流感染、感染性心内膜炎、化脓性脑膜炎、伤寒、布鲁菌病、骨髓炎、B组链球菌咽炎和扁桃体炎等需较长的疗程[25],[39]。

2. 病毒感染的治疗：病毒感染需针对性用药。疱疹病毒推荐阿昔洛韦或伐昔洛韦（疗程7～10 d[40]）；CMV DNA水平连续2次>500拷贝/ml或单次>1 000拷贝/ml可作为启动抢先治疗的标准，药物推荐缬更昔洛韦、更昔洛韦和膦甲酸钠等（建议每周检测CMV-DNA，连续2次阴性后可停药）[41]–[42]；HBV慢性感染：可选择恩替卡韦或替诺福韦等治疗（具体疗程建议咨询感染科专家）；EB病毒感染尚无统一治疗方案，以B细胞受累为主的EB病毒感染可使用利妥昔单抗治疗[26]。新型冠状病毒感染推荐使用奈玛特韦/利托那韦（连续5 d）、莫诺拉韦（连续5 d）或阿兹夫定（不超过14 d）等药物[28],[43]。用药前核对合并用药的相互作用及肾功能剂量调整，必要时选择替代方案。

3. 真菌感染的治疗：明确病原体后可依据真菌种类、药物抗菌谱、患者具体情况选择药物（表3）[32],[44]–[45]。真菌治疗疗程根据真菌证据确定，至少应用至体温降至正常、临床状况稳定；诊断驱动治疗应包括侵袭性真菌病相关微生物学和（或）影像学指标恢复正常[31]。治疗期间建议暂停使用BsAb，直至患者症状消退[26]。

**表3:** 接受双特异性抗体治疗的多发性骨髓瘤患者抗真菌治疗药物选择

类型	首选/一线推荐用药	备注
侵袭性念珠菌病[44]	卡泊芬净、米卡芬净	非重症和无唑类药物暴露患者也可选择唑类药物；念珠菌败血症不伴粒细胞缺乏患者中，初始治疗病情稳定、血培养转阴5～7 d后（初始治疗10 d以上）可改用静脉或口服唑类药物治疗，危重症等免疫力极度低下患者初始治疗的疗程相应延长。念珠菌败血症合并粒细胞缺乏且无播散性病灶患者，应血培养转阴后至少再治疗2周，且感染征象消失、粒细胞缺乏恢复
侵袭性曲霉病[31]	伏立康唑、艾沙康唑	推荐6～12周，根据侵袭性曲霉病临床严重程度、相关症状和体征恢复速度以及免疫抑制状态改善情况决定停药时机
毛霉病[45]	艾沙康唑、两性霉素B脂质体	治疗至少3～6个月，直到免疫抑制状态永久逆转，影像学表现提示完全缓解[46]–[47]
耶氏肺孢子菌肺炎[44]	复方磺胺甲噁唑	若对复方磺胺甲噁唑过敏或葡萄糖-6-磷酸脱氢酶缺乏者，可选择阿托伐醌或伯氨喹＋克林霉素[26]。疗程推荐21 d

4. 结核分枝杆菌感染的治疗：初治活动性肺结核（含痰涂片阳性和阴性）通常选用2HRZE/4HR方案，即强化期使用异烟肼（H）、利福平（R）、吡嗪酰胺（Z）、乙胺丁醇（E），1次/d，共2个月；巩固期使用异烟肼、利福平，1次/d，共4个月。若强化期第2个月末痰涂片仍阳性，强化方案可延长1个月，总疗程6个月不变[48]。

复治活动性肺结核（含痰涂片阳性和阴性）：常用方案为2HRZSE/6HRE、3HRZE/6HR和2HRZSE/1HRZE/5HRE，“S”为链霉素。

对至少包括异烟肼和利福平在内的2种以上药物产生耐药的结核为多药耐药结核，可选择氟喹诺酮类［大剂量左氧氟沙星（≥750 mg/d）、莫西沙星及加替沙星］、阿米卡星、卷曲霉素等[48]。抗结核治疗应在胸科医院/感染科专家指导下进行。

**推荐意见**

1. BsAb治疗期间发生感染后，应在综合评估患者基础状况、感染部位、脏器功能、耐药危险因素及当地病原流行病学的基础上，尽快启动经验性抗感染治疗，必要时进行多学科诊疗，并注意药物相互作用及肾功能相关剂量调整。

2. 细菌感染治疗应尽可能依据病原学及药敏结果选择抗菌药物；对于病原体未明，尤其是合并中性粒细胞减少者，应及时给予广谱抗菌药物经验性治疗。

3. 病毒和真菌感染应根据病原学结果及临床情况选择针对性治疗；真菌感染治疗期间建议暂停BsAb，直至患者症状消失。

4. 感染相关BsAb治疗调整应根据其严重程度决定：1～2级感染经积极处理和密切监测后可继续治疗，3级感染需暂停，4级感染考虑永久停药；3～4级发热伴中性粒细胞减少也应暂停，待感染改善至相应恢复标准后再恢复用药。

BsAb治疗MM相关感染监测及防控流程见图1。

**图1:**
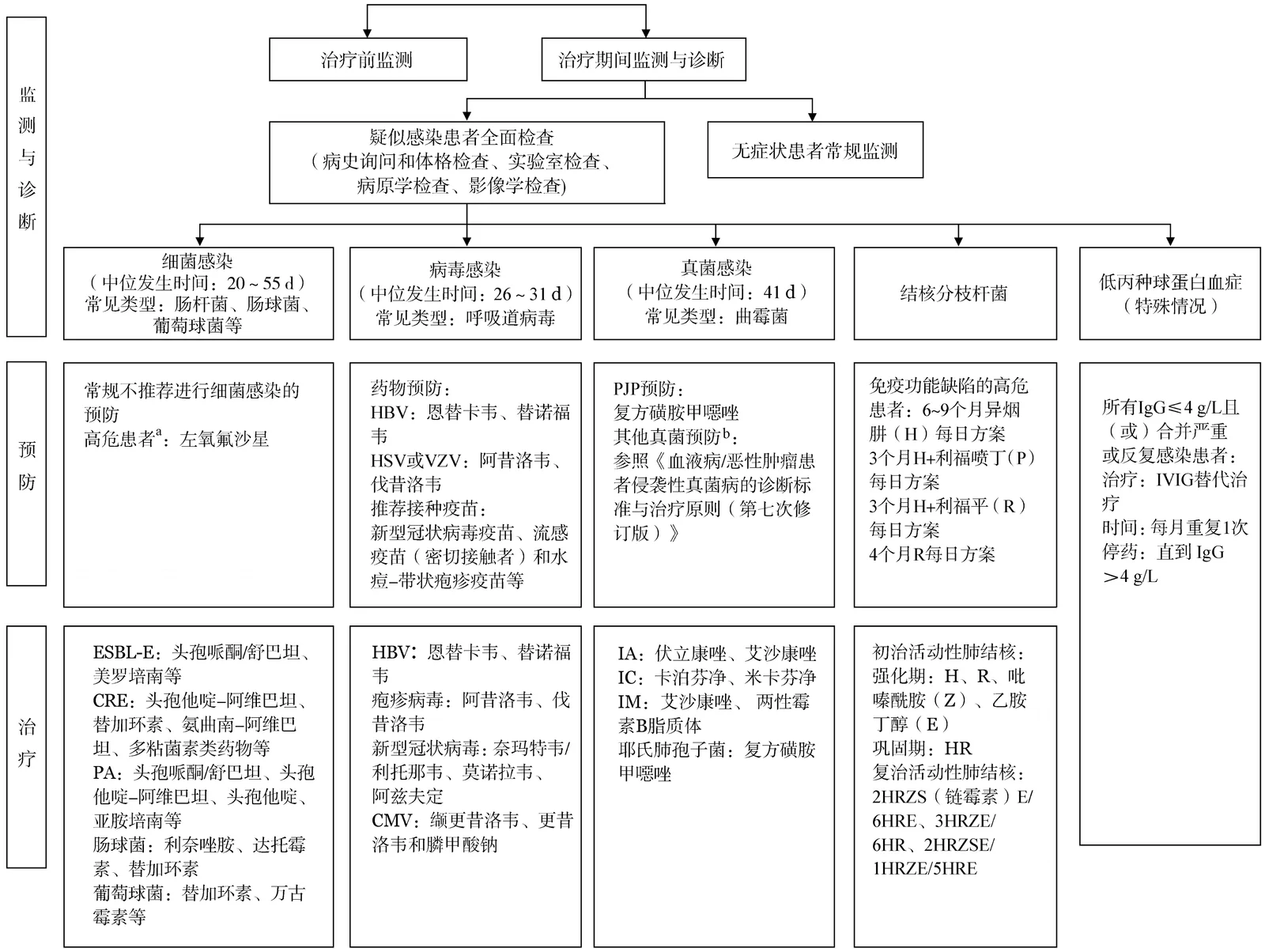
双特异性抗体治疗多发性骨髓瘤相关感染监测及防控流程图 **注** HBV：乙型肝炎病毒；HSV：单纯疱疹病毒；VZV：水痘-带状疱疹病毒；PJP：耶氏肺孢子菌肺炎；ESBL-E：产超广谱β-内酰胺酶肠杆菌科细菌；CRE：碳青霉烯类耐药的肠杆菌科细菌；PA：铜绿假单胞菌；CMV：巨细胞病毒；IA：侵袭性曲霉病；IC：侵袭性念珠菌病；IM：侵袭性毛霉病；IVIG：静脉注射免疫球蛋白；^a^存在细菌感染史/复发性细菌感染史、已知的病原体定植或长期中性粒细胞减少；^b^具有侵袭性真菌病高危因素的患者，如异基因造血干细胞移植患者、急性白血病或骨髓增生异常综合征接受初次诱导或挽救化疗患者、预计中性粒细胞减少持续>10 d患者、伴有严重中性粒细胞缺乏或接受抗胸腺球蛋白治疗或造血干细胞移植的重症再生障碍性贫血患者等

六、结语

BsAb是RRMM的重要治疗方案，其相关感染需高度重视。加强对BsAb相关感染的防控不仅是保障治疗安全的关键，也是改善患者预后、提升治疗价值的重要环节。本共识聚焦于MM患者接受BsAb治疗这一特定临床场景，强调在MM专病背景下开展感染风险识别、监测、预防和干预。随着BsAb在我国MM领域的进一步应用，临床实践中积累的经验将为优化相关感染防治策略提供更具针对性的依据。

未来，BsAb相关感染的预防与管理可朝着精准化、高效化方向发展。一方面，需进一步优化BsAb的给药剂量、间隔及整体治疗方案，基于真实世界数据构建兼具科学性与实用性的感染风险预测模型，为不同分层风险人群制定个体化防控策略；另一方面，应升级感染监测与诊断体系，加速快速病原检测技术的临床转化与推广应用，缩短感染识别与确诊的时间窗口。同时，探索适配BsAb治疗周期的全程感染防控方案，健全多学科协作机制，在最大限度保障患者安全的前提下，充分提升BsAb的治疗效能。随着其他血液系统恶性肿瘤中BsAb应用证据的不断积累，未来可进一步针对不同病种形成更具适用性的感染管理建议。
